# Real-world outcomes of third-line immune checkpoint inhibitors versus irinotecan-based chemotherapy in patients with advanced gastric cancer: a Korean, multicenter study (KCSG ST22-06)

**DOI:** 10.1186/s12885-024-11972-w

**Published:** 2024-02-23

**Authors:** Sung Hee Lim, Keun-Wook Lee, Jae-Joon Kim, Hyeon-Su Im, In-Ho Kim, Hye Sook Han, Dong-Hoe Koo, Jang Ho Cho, Chi Hoon Maeng, Min-Young Lee, Hyo Jin Lee, Jwa Hoon Kim, Sang Gon Park, Joo Young Jung, Seong-Hoon Shin, Ki Hyang Kim, Hyeyeong Kim, So Yeon Oh, Minsu Kang, Minkyu Jung, Sun Young Rha

**Affiliations:** 1 Division of Hematology-Oncology, Department of Internal Medicine, Samsung Medical Center, Seoul, South Korea; 2 Division of Hematology-Oncology, Department of Internal Medicine, Soonchunhyang University Bucheon Hospital, Bucheon, South Korea; 3 Department of Internal Medicine, Seoul National University Bundang Hospital, Seoul National University College of Medicine, Seongnam, Korea; 4 Department of Internal Medicine, School of Medicine, Pusan National University Yangsan Hospital, Pusan National University, Yangsan, Republic of Korea; 5 Department of Hematology and Oncology, Ulsan University Hospital, Ulsan University College of Medicine, Ulsan, South Korea; 6 Division of Oncology, Department of Internal Medicine, Seoul St. Mary’s Hospital, College of Medicine, The Catholic University of Korea, Seoul, South Korea; 7 Department of Internal Medicine, Chungbuk National University Hospital, Chungbuk National University College of Medicine, Cheongju, South Korea; 8 Department of Internal Medicine, Kangbuk Samsung Hospital, Sungkyunkwan University School of Medicine, Seoul, South Korea; 9 Division of Oncology, Department of Internal Medicine, Incheon St. Mary’s Hospital, College of Medicine, The Catholic University of Korea, Seoul, South Korea; 10 Division of Hematology-Oncology, Department of Internal Medicine, College of Medicine, Kyung Hee University, Seoul, South Korea; 11 Division of Hematology-Oncology, Department of Internal Medicine, Soonchunhyang University Seoul Hospital, Seoul, South Korea; 12 Division of Hemato-Oncology, Department of Internal Medicine, Chungnam National University Hospital, Daejeon, South Korea; 13 Department of Internal Medicine, Anam Hospital, Korea University College of Medicine, Seoul, South Korea; 14 Department of Hemato-Oncology, Chosun University Hospital, Gwangju, South Korea; 15 Division of Hemato-Oncology, Hallym University Dongtan Sacred Heart Hospital, Hwaseong, South Korea; 16 Department of Hemato/Oncology, Internal Medicine, Kosin University Gospel Hospital, Busan, South Korea; 17 Division of Oncology, Department of Internal Medicine, Inje University College of Medicine, Busan Paik Hospital, Busan, South Korea; 18 Division of Medical Oncology, Department of Internal Medicine, Yonsei Cancer Center, Yonsei University Health System, Seoul, South Korea

**Keywords:** Gastric cancer, Third-line treatment, Irinotecan, Nivolumab, Pembrolizumab

## Abstract

**Background:**

Immune checkpoint inhibitor (ICI) or irinotecan-based chemotherapy is frequently used after failure of second-line paclitaxel plus ramucirumab treatment for patients with locally advanced unresectable or metastatic advanced gastric cancer (AGC). This study aimed to compare the efficacy between ICI and irinotecan-based chemotherapy as third-line treatment in patients with AGC.

**Methods:**

We retrospectively reviewed patients with AGC, whose third-line treatment started between July 2019 and June 2021 at 17 institutions in Korea. The ICI group included patients who received nivolumab or pembrolizumab, and the irinotecan-based chemotherapy group included patients who received irinotecan or FOLFIRI (5-fluorouracil, leucovorin and irinotecan).

**Results:**

A total of 363 patients [*n* = 129 (ICI) and *n* = 234 (irinotecan-based chemotherapy)] were analyzed. The median progression-free survival was 2.3 and 2.9 months in ICI and irinotecan-based chemotherapy groups, respectively (*p* = 0.802). The median overall survival (OS) was 5.5 and 6.0 months in ICI and irinotecan-based chemotherapy groups, respectively (*p* = 0.786). For all patients included in this study, multivariable analysis showed that weight loss, peritoneal metastasis, low serum sodium or albumin, and short duration of second-line treatment were associated with inferior OS (*p* < 0.05). ICI showed significantly longer OS than irinotecan-based chemotherapy in patients without peritoneal metastasis. Whereas ICI showed significantly shorter OS in patients without PD-L1 expression than irinotecan-based chemotherapy.

**Conclusions:**

No significant difference in survival outcome was observed between ICI and irinotecan-based chemotherapy as third-line treatment for AGC patients. ICI might be preferred for patients without peritoneal metastasis and irinotecan-based chemotherapy for patients with tumors without PD-L1 expression.

**Trial registration:**

This study was registered in the Clinical Trial Registry of Korea (https://cris.nih.go.kr: KCT 0007732).

**Supplementary Information:**

The online version contains supplementary material available at 10.1186/s12885-024-11972-w.

## Background

Gastric cancer (GC) is the fifth most diagnosed malignancy and the third most common cause of cancer mortality globally. In South Korea, the incidence of GC is the second most common cancer and the fourth leading cause of cancer-related death [1]. The prognosis of locally advanced unresectable or metastatic advanced gastric cancer (AGC) is poor, with a median overall survival (OS) of approximately one year. Fluoropyrimidine/platinum doublet chemotherapy has been recommended as the standard first-line treatment for most patients with AGC and adding trastuzumab is strongly recommended for patients with human epidermal growth factor receptor 2 (HER2)-positive GC. Recently, it was shown that addition of immune checkpoint inhibitor (ICI) to fluoropyrimidine/platinum is associated with superior OS compared to fluoropyrimidine/platinum alone in patients with AGC. Consequently, the addition of nivolumab, an anti-programmed death-1 (anti-PD-1) monoclonal antibody, to fluoropyrimidine/platinum has become a new first-line standard of care in AGC with programmed death-ligand 1 (PD-L1) expression levels of ≥ 5 in terms of combined positive score (CPS) based on the CHECKMATE 649 trial [2]. Additionally, another anti-PD-1 antibodies (pembrolizumab or tislelizumab), when combined with fluoropyrimidine/platinum, showed superior survival outcomes compared to chemotherapy alone [3, 4].

For second-line treatment, paclitaxel plus ramucirumab is the most commonly used regimen based on a significant improvement in OS compared to paclitaxel monotherapy in the RANIBOW trial [5, 6].

After discontinuation of second-line ramucirumab plus paclitaxel, third-line systemic chemotherapy was administered to 47% of all patients in a Korean nationwide real-world study [7]. Among several treatment options proven to be effective in third-line treatment settings, regimens are selected in consideration of prior therapy, disease burden, and the patient’s condition including performance status (PS). Trifluridine/tipiracil showed significant improvements in OS compared with placebo in patients with heavily treated AGC and was approved by the US FDA in 2019 [8]. In the phase 3 randomized ATTRACTION-2 trial, Asian patients treated with nivolumab as ≥ third-line treatment reported a significant improvement in OS compared to patients with placebo [9]. In the international phase 2 non-randomized KEYNOTE-059 trial, pembrolizumab showed similar OS in patients with refractory AGC [10]. Irinotecan-based chemotherapy [irinotecan monotherapy or 5-fluorouracil, leucovorin and irinotecan (FOLFIRI)] can be a preferred treatment option in the third-line treatment setting after the use of fluoropyrimidine, platinum, and taxane [11–13]. As nivolumab combined with chemotherapy has become a new first-line standard treatment for AGC, especially in tumors with PD-L1 expression, a change is expected in the positioning of PD-1 inhibitors, which have been frequently used as the third or later line treatment. However, ICI will still be an important third or later-line treatment option in patients with ICI-naïve tumors with no/low PD-L1 expression or with HER2-positive or claudin 18.2-positive tumors [14–16].

In this study, we conducted a multi-center, real-world study to compare the effectiveness between anti-PD-1 therapy (nivolumab or pembrolizumab) and irinotecan-based cytotoxic chemotherapy, which are the most commonly used as third-line treatment regimens, for patients with AGC who failed second-line paclitaxel plus ramucirumab treatment.

## Methods

### Patients

This retrospective multicenter (17 tertiary referral centers in Korea) real-world study was conducted by the stomach cancer committee of the Korean Cancer Study Group (study number: KCSG ST22-06). Enrolled patients were those with histologically or cytologically confirmed gastric or gastroesophageal junction adenocarcinoma who received either ICI (nivolumab or pembrolizumab) or irinotecan-based chemotherapy (irinotecan or FOLFIRI) as third-line treatment after failure of second-line paclitaxel plus ramucirumab therapy. In this study, only patients who started third-line treatment between July 2019 and June 2021 were selected and their medical records were reviewed. Patients who had recurrence within 6 months after the completion of adjuvant chemotherapy [capecitabine and oxaliplatin (XELOX) or S-1)] were considered to have failed first-line treatment. This study was approved by the institutional review board (IRB) of each institution as required. Due to the retrospective nature of this study, the requirement for informed consent was waived by the IRBs of all participating institutions, and the names of the IRBs of each institution related to this are described in ‘Ethics approval and consent to participate’. This study was registered in the Clinical Trial Registry of Korea (https://cris.nih.go.kr: KCT 0007732).

### Treatment and assessment

The ICI treatment group included patients who received nivolumab or pembrolizumab. Nivolumab was administered intravenously at a dose of 3 mg/kg every 2 weeks [9] and pembrolizumab was administered intravenously at a fixed dose of 200 mg every 3 weeks [10]. Irinotecan was administered intravenously at a dose of 150 mg/m^2^ every 2 weeks [12]. FOLFIRI consisted of intravenous infusion of irinotecan at a dose of 150–180 mg/m^2^ followed by leucovorin at a dose of 400 mg/m^2^, and a bolus of 5-fluorouracil at a dose of 400 mg/m^2^ with a continuous infusion of 5-fluorouracil at a dose of 2400 mg/m^2^ (over 46 h) every 2 weeks, in line with previous studies [11, 13, 17]. Dose adjustments and reductions were made at the discretion of the attending physician in consideration of patient’s characteristics such as elderliness, reduced PS, comorbidity, or depending on the degree of previous treatment-related toxicity. Treatment was continued until progressive disease (PD) or intolerable toxicity. Computed tomography [CT; abdomen/pelvis CT ± chest CT] or magnetic resonance imaging (MRI; if CT scan could not be performed) were carried out every 6–8 weeks. Adverse events (AEs) were monitored at every clinic visit. Tumor response and AEs were graded by Response Evaluation Criteria In Solid Tumor (RECIST, version 1.1) and National Cancer Institute-Common Terminology Criteria for Adverse Events (NCI-CTCAE) version 4.03, respectively.

### Statistical analysis

Progression-free survival (PFS) was defined as the time from treatment initiation to PD or any cause of death, whichever occurred first. OS was defined as the time from treatment initiation to any cause of death. Data cut-off for survival analysis was set at Dec 31, 2021. Pearson’s chi-square test or Fisher’s exact test was used to compare discrete data. Survival outcomes were estimated using the Kaplan-Meier method and compared using the log-rank test. Univariable and multivariable analyses were performed using the Cox proportional hazard model. A two-sided p-value < 0.05 was considered statistically significant. All statistical analyses were carried out using Statistical Package for the Social Sciences for Windows (version 27.0; IBM Corp. Armonk, NY, USA).

## Results

### Patient characteristics

A total of 363 patients were included in this study [ICI group (*n* = 129) and irinotecan-based chemotherapy group (*n* = 234)]. In the ICI group, 96 patients (74%) were treated with nivolumab while 33 patients (26%) with pembrolizumab. In the irinotecan-based chemotherapy group, 99 patients (42%) received irinotecan monotherapy while 135 patients (58%) were treated with FOLFIRI. The median treatment duration of second-line paclitaxel plus ramucirumab was 4.1 months in the ICI group and 3.5 months in the irinotecan-based chemotherapy group. There was no significant difference in the patient characteristics between the two groups except for PD-L1 status and microsatellite instability (MSI)/mismatch repair (MMR) status (Table 1). Data on HER2, Epstein-Barr virus (EBV), MMR or MSI and PD-L1 were available in 358, 280, 281 and 239 patients, respectively. HER2-positive tumors were found in 18 patients (14%) in the ICI group and 26 patients (11%) in the irinotecan-based chemotherapy group. EBV-positive tumors were observed in the two of 98 patients (2%) in the ICI group and 11 of 182 patients (6%) in the irinotecan-based chemotherapy group. The proportion of PD-L1 positive GC was higher in the ICI group patients than those treated with irinotecan-based chemotherapy (62% versus 39%, *p* = 0.001). PD-L1 expression was assessed using immunohistochemical (IHC) staining with either tumor proportion score (TPS) or CPS, depending on each institution’s test performance and diagnosis policy and PD-L1 positivity was defined as CPS or TPS ≥ 1% in this study. Therefore, PD-L1-negative tumors were defined as CPS < 1% or TPS < 1%. MSI-high (MSI-H) and/or MMR-deficient (dMMR) tumors were reported in nine of 99 patients (9%) in the ICI group whereas five out of 182 (3%) in the irinotecan-based chemotherapy group (*p* = 0.024).

**Table 1:** Baseline characteristics of patients

	Immune checkpoint inhibitor group (*n* = 129)	Irinotecan-based chemotherapy group (*n* = 234)	P-value
	No. (%)	No. (%)	
**Age, years**			0.099
Mean ± SD	59 ± 11.04	57 ± 11.45	
Range	22–88	26–83	
**Sex**			0.682
Male	76 (58.9)	143 (61.1)	
Female	53 (41.1)	91 (38.9)	
^a^ **ECOG performance status**			0.469
0/1	31 (24.2)/ 68 (53.1)	40 (17.2)/ 166 (71.6)	
2/3	28 (21.9)/ 1 (0.8)	25 (10.8)/ 1 (0.4)	
^b^ **Site of primary tumor**			0.234
Gastric	119 (97.5)	202 (94.8)	
Gastroesophageal junction	3 (2.5)	11 (5.2)	
^c^ **WHO histology**			0.881
Tubular adenocarcinoma			
Well differentiated	3 (2.3)	7 (3.0)	
Moderately differentiated	35 (27.1)	61 (26.1)	
Poorly differentiated	33 (25.6)	63 (26.9)	
Poorly cohesive carcinoma/Signet-ring cell carcinoma	50 (38.8)	88 (37.6)	
Mixed adenocarcinoma	6 (4.7)	7 (3.0)	
^d^ **Lauren Classification**			0.320
Intestinal	28 (32.2)	35 (28.7)	
Diffuse	52 (59.8)	71 (58.2)	
Mixed	7 (8.0)	16 (13.1)	
**Measurable lesion**			0.581
Yes	75 (58.1)	128 (54.7)	
No	54 (41.9)	106 (45.3)	
^e^ **HER2 status**			0.446
Positive	18 (14.1)	26 (11.3)	
Negative	110 (85.9)	204 (88.7)	
^f^ **EBV in situ hybridization**			0.129
Positive	2 (2.0)	11 (6.0)	
Negative	96 (98.0)	171 (94.0)	
^g^ **MSI/MMR status**			0.024
MSI-H and/or dMMR	9 (9.1)	5 (2.7)	
Others	90 (90.9)	177 (97.3)	
^h^ **PD-L1 status**			0.001
Positive	54 (62.1)	60 (39.4)	
Negative	33 (37.9)	92 (60.5)	
^i^ **Prior gastrectomy**			0.303
Yes	74 (57.4)	120 (51.7)	
No	55 (42.6)	112 (48.3)	
**Peritoneal metastasis**			0.262
Yes	90 (69.8)	176 (75.2)	
No	39 (30.2)	58 (24.8)	
**Liver metastasis**			0.089
Yes	35 (27.1)	84 (35.9)	
No	94 (72.9)	150 (64.1)	
**Duration of 2nd line treatment**			0.841
Median (range), months	4.1 (0.6–23.0)	3.5 (0.3–25.4)	
**Prior first-line treatment**			0.305
Trastuzumab plus FP (or XP)	16 (12.4)	21 (9.0)	
XELOX or FOLFOX	96 (74.4)	167 (71.4)	
XP or SP	12 (9.3)	24 (10.3)	
Others*	5 (3.8)	22 (9.4)	
^j^ **Serum sodium, mEq/L**			
Median (range)	138.0 (127.0-146.0)	138.4 (121.0-149.0)	
≥135	89 (73.0)	172 (81.9)	0.071
<135	33 (27.0)	38 (18.1)	
^k^ **Serum albumin, g/dL**			
Median (range)	3.3 (1.8–4.6)	3.4 (1.9–4.6)	
≥3.5	50 (39.7)	104 (45.4)	0.315
<3.5	76 (60.3)	125 (54.6)	

Abbreviations: ECOG, Eastern Cooperative Oncology Group; WHO, World Health Organization; HER2, human epidermal growth factor receptor 2; EBV, Epstein-Barr virus; MSI/MMR, microsatellite instability/mismatch repair; FP, 5-fluorouracil and cisplatin; XP, capecitabine and cisplatin; XELOX, capecitabine and oxaliplatin; FOLFOX, 5-fluorouracil, leucovorin, and oxaliplatin; SP, S-1 and cisplatin; SOX, S-1 and oxaliplatin; FEP, 5-fluorouracil, etoposide and cisplatin

^a^Missing ECOG PS data for 3 patients; ^b^Missing site of primary tumor for 7 in the ICI and 21 in the irinotecan-based chemotherapy grup; ^c^Missing WHO histology for 2 in the ICI and 8 in the irinotecan-based chemotherapy group; ^d^Missing Lauren classification for 42 in the ICI and 112 in the irinotecan-based chemotherapy group; ^e^Missing HER2 status for 1 patient in the ICI and 4 patients in the irinotecan-based chemotherapy group; ^f^Missing EBV status for 31 patients in the ICI and 52 patients in the irinotecan-based chemotherapy group; ^g^Missing MSI/MMR status for 30 patients in the ICI and 52 patients in the irinotecan-based chemotherapy group; ^h^Missing PD-L1 status for 42 patients in the ICI and 82 patients in the irinotecan-based chemotherapy group; ^i^Missing prior gastrectomy for 2 in the irinotecan-based chemotherapy group; ^j^Missing serum sodium for 7 patients in the ICI and 24 patients in the irinotecan-based chemotherapy group; ^k^Missing serum albumin for 3 in the ICI and 5 in the irinotecan-based chemotherapy group

*Others include SOX, FP, FEP, and clinical trials (FOLFOX+/-Zolbetuximab, FOLFOX+/-FPA144, XELOX+/-Nivolumab, XELOX+/-BGB-A317, FOLFOX+/-Varlitinib, SOX+/-Nivolumab)

### Treatment effectiveness

**Fig. 1:**
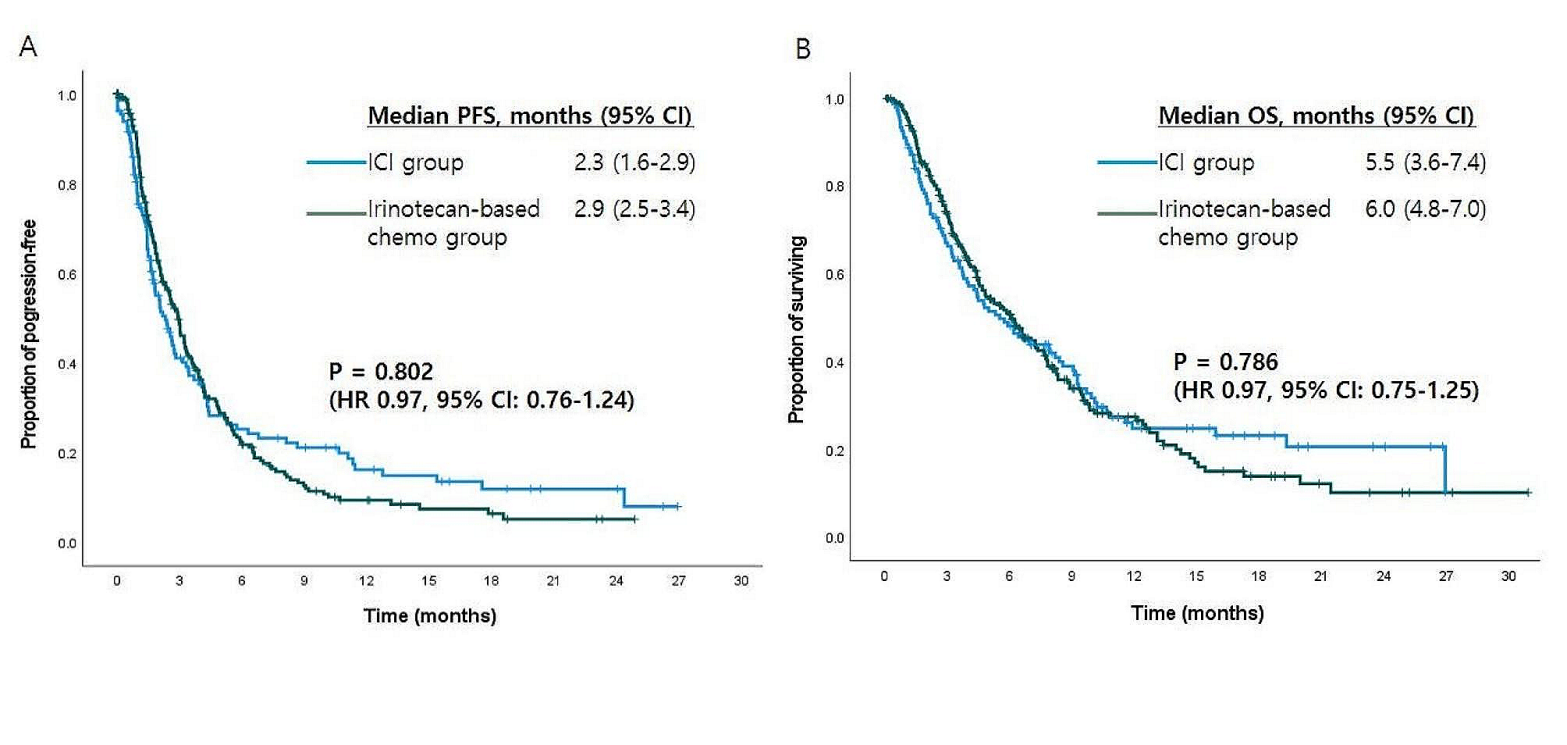
Kaplan-Meier survival curves by treatment arm (**A**) Progression-free survival (PFS) and (**B**) Overall survival (OS). (ICI, immune checkpoint inhibitor; HR, hazard ratio; CI, confidence interval)

Data cut-off for survival analysis was set at December 31, 2021. At the data cut-off date, 283 (78%) PFS events had occurred and 253 patients (70%) had died. The median follow-up duration for all patients was 12.6 months (range, 0.1–30.9). There was no significant difference in median PFS between the two groups: 2.3 months [95% confidence interval (CI), 1.6–2.9] in patients treated with ICI and 2.9 months (95% CI, 2.5–3.4) in patients treated with irinotecan-based chemotherapy [hazard ratio (HR) 0.97 (95% CI, 0.76–1.24); *p* = 0.802; Fig. 1A]. The median OS was 5.5 months (95% CI, 3.6–7.4) in the ICI group and 6.0 months (95% CI, 4.8-7.0) in the irinotecan-based chemotherapy group [HR 0.97 (95% CI, 0.75–1.25); *p* = 0.786; Fig. 1B]. The 12-month and 24-months OS rates were respectively 25% and 20% in the ICI group compared with 27% and 10% in the irinotecan-based chemotherapy group.

**Fig. 2:**
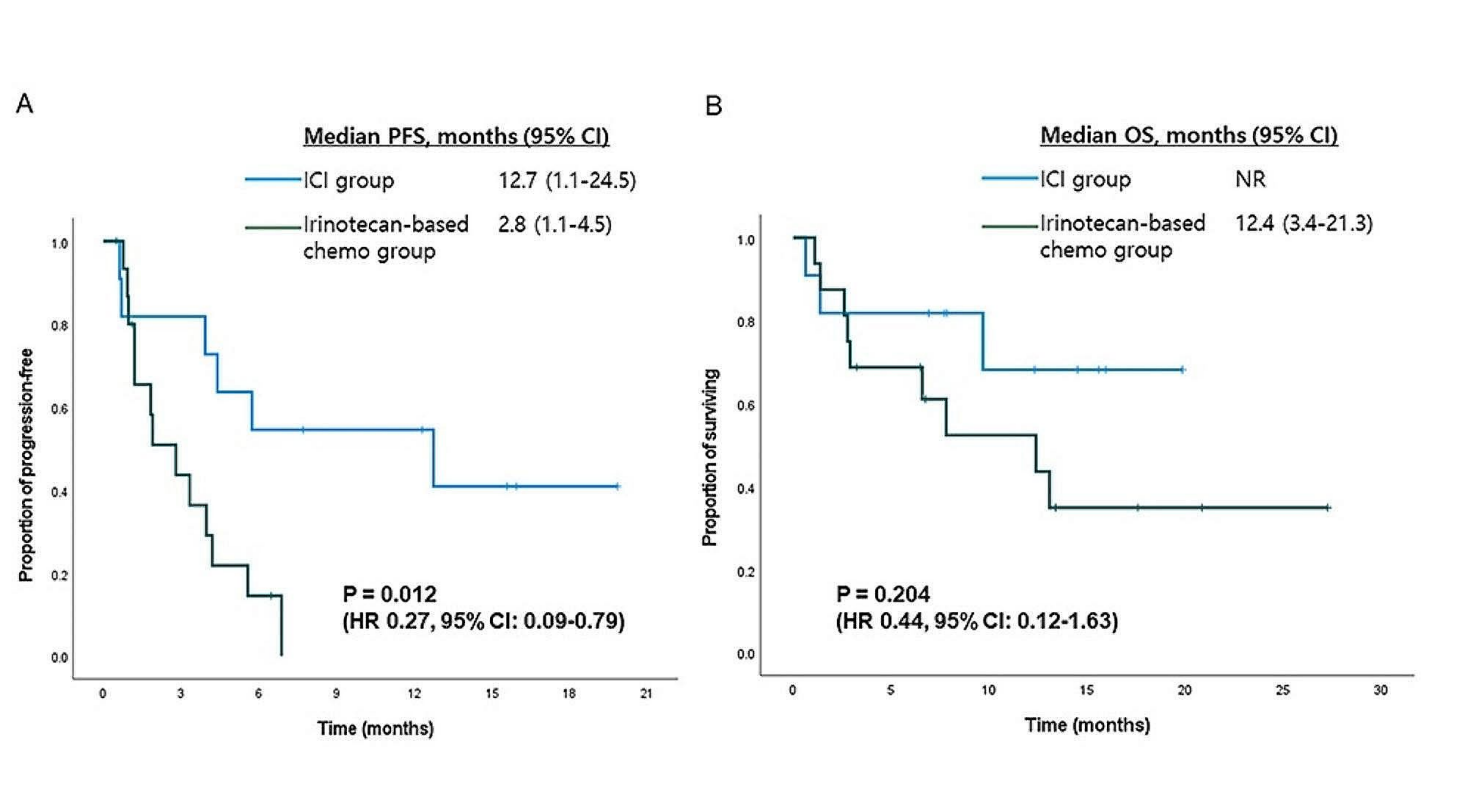
Kaplan-Meier survival curves by treatment arm in patients with MSI-H/dMMR or EBV-positive tumors. (**A**) Progression-free survival (PFS) and (**B**) Overall survival (OS). (ICI, immune checkpoint inhibitor; HR, hazard ratio; CI, confidence interval)

There were 27 patients whose tumors were either MSI-H/dMMR (*n* = 14) or EBV-positive (*n* = 13). ICI treatment was associated with significantly longer PFS in this patient subset than irinotecan-based chemotherapy. The median PFS was 12.7 months (95% CI, 1.1–24.5) in the ICI group and 2.8 months (95% CI, 1.1–45) in the irinotecan-based chemotherapy group [HR 0.27 (95% CI, 0.09–0.79); *p* = 0.012; Fig. 2A]. The median OS was not reached in the ICI group while it was 12.4 months in the irinotecan-based chemotherapy group; however, there was no statistical difference between the two groups [HR 0.44 (95% CI 0.12–1.63); *p* = 0.204; Fig. 2B]. Detailed clinical characteristics and treatment outcomes in patients with MSI-H/dMMR or EBV-positive tumor are listed in Supplementary Table S1.

Additionally, the survival outcomes of ICI versus irinotecan-based chemotherapy as third-line treatment for patients with PD-L1-negative and/or HER2-positive tumors (*n* = 157) was analyzed. In this subgroup, ICI and irinotecan-based chemotherapy did not show significant differences in PFS and OS (Supplementary Figure S1). The median PFS was 2.0 months (95% CI, 1.1-3.0) in patients treated with ICI and 2.5 months (95% CI, 1.8–3.3) with irinotecan-based chemotherapy (*p* = 0.612). The median OS was 4.4 months (95% CI, 3.3–5.5) in the ICI group and 6.3 months (95% CI, 3.5-9.0) in the irinotecan-based chemotherapy group (*p* = 0.224).

Objective response rate (ORR) was higher in patients treated with ICI than in patients treated with irinotecan-based chemotherapy (*p* = 0.038) (Table 2). Overall, four patients and 11 patients in the ICI group, respectively, had complete response (CR) and partial response (PR), resulting in ORR of 12%. In the irinotecan-based chemotherapy group, one patient had CR and 12 patients had PR, resulting in ORR of 6%. Disease control was achieved in 45 patients in the ICI group (35%) and 105 patients in the irinotecan-based chemotherapy group (45%) (*p* = 0.064).

**Table 2:** Best overall response in the overall population

Response	Immune checkpoint inhibitor (*n* = 129)		Irinotecan-based chemotherapy (*n* = 234)		
	No.	%	No.	%	P value
CR	4	3.1	1	0.4	
PR	11	8.5	12	5.1	
SD^a^	30	23.3	92	39.3	
PD	67	51.9	107	45.7	
NE	17	13.2	22	9.4	
Objective response rate^b^	15	11.6	13	5.6	0.038
Disease control rate^c^	45	34.9	105	44.9	0.064

Abbreviations: CR, complete response; PR, partial response; SD, stable disease; PD, progressive disease; NE, not evaluable

^a^Non-CR/non-PD for cases without measurable disease were included in SD. ^b^Objective response rate is defined as the proportion of patients with CR or PR as best overall response. ^c^Disease control rate is CR + PR + SD (including nonCR/non-PD for cases with non-measurable disease only) as the best response

Univariable and multivariable analyses of PFS and OS were performed for all patients included in this study (Supplementary Table S2 and Table 3 ). The multivariable analysis identified poor Eastern Cooperative Oncology Group (ECOG) PS (grade 2 or 3) and 10% or more weight loss within 3 months before starting third-line treatment as independent poor prognostic factors for PFS. Meanwhile, weight loss, peritoneal metastasis, low serum sodium (< 135 mEq/L), low serum albumin (< 3.5 g/dL), and short duration of second-line treatment (< median) were identified as independent prognostic factors for worse OS. MSI-H/dMMR status was an independent favorable prognostic factor for PFS and OS (Table 3).

**Table 3:** Multivariable analysis of prognostic factors

	PFS			OS		
	HR	95% CI	P value	HR	95% CI	P value
**ECOG PS**						
2/3 versus 0/1 (Ref)	2.27	1.25–4.10	0.007	-	-	-
**Primary site**						
GEJ versus Stomach (Ref)	-	-	-	-	-	-
**Weight loss**						
Yes versus No (Ref)	1.67	1.21–2.28	0.001	1.46	1.04–2.01	0.030
**Peritoneal seeding**						
Yes versus No (Ref)	-	-	-	1.76	1.10–2.80	0.019
**MSI/MMR status**						
MSI-H/dMMR versus others (Ref)	0.23	0.10–0.53	0.001	0.03	0.004–0.21	< 0.001
**Serum sodium, mEq/L**						
< 135 versus ≥ 135 (Ref)	-	-	-	1.92	1.24–2.96	0.003
**Serum albumin, g/dL**						
< 3.5 versus ≥ 3.5 (Ref)	-	-	-	1.78	1.19–2.66	0.005
**Duration of 2nd line therapy** ^**a**^						
<median versus ≥ median (Ref)	-	-	-	2.17	1.53–3.08	< 0.001

Abbreviations: PFS, progression-free survival; OS, overall survival; HR, hazard ratio; CI, confidence interval; Ref, reference; ECOG, Eastern Cooperative Oncology Group; PS, performance status; GEJ, gastroesophageal junction; MSI/MMR, microsatellite instability/mismatch repair

^a^The median duration of 2nd line treatment was 3.7 months

*Univariable analyses included potential prognostic factors for predicting survival associated with ICI; age, ECOG PS, weight loss in the previous 3 months, presence of liver metastasis, presence of peritoneal metastasis, prior gastrectomy, neutrophil to lymphocyte ratio (NLR), platelet to lymphocyte ratio (PLR), serum sodium, serum albumin, primary site, duration of 2nd line paclitaxel/ramucirumab treatment, MSI/MMR status, HER2 status, EBV status

**Multivariable analysis was performed using a backward selection method for factors that showed significant results in the univariable analyses

**Fig. 3:**
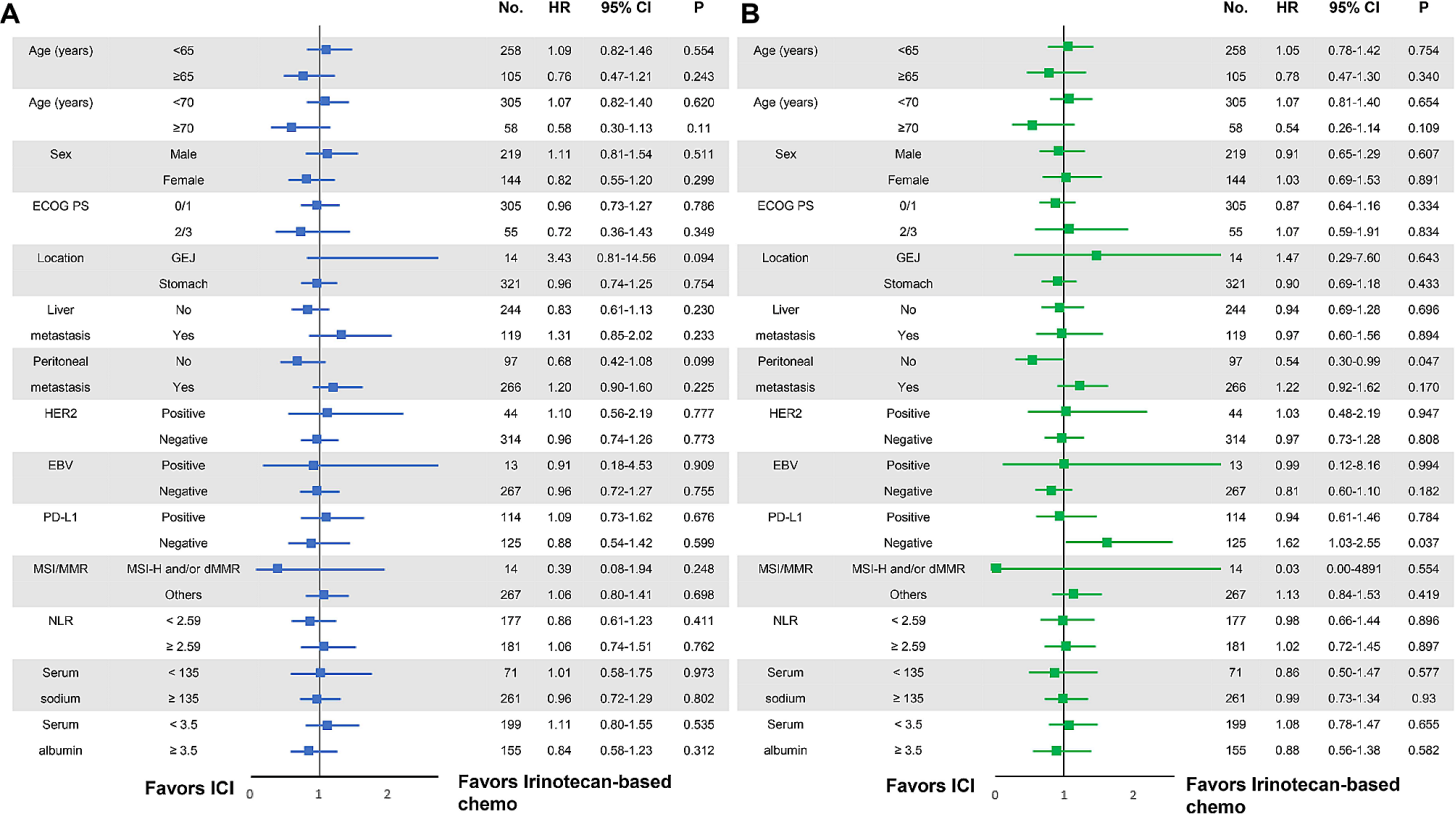
Forrest plots showing the survival outcomes of patient subgroups. (A) Progression-free survival (PFS) and (B) Overall survival (OS). (HR, hazard ratio; CI, confidence interval; ECOG, Eastern Cooperative Oncology Group; PS, performance status; GEJ, gastroesophageal junction; NLR, neutrophil to lymphocyte ratio)

Although not statistically significant in subgroup analyses, patients without peritoneal metastasis showed a trend of better PFS with ICI than irinotecan-based chemotherapy [HR 0.68 (95% CI, 0.42–1.08); *p* = 0.099). Subgroup analyses revealed a significant difference in OS between patients with and without peritoneal metastasis. ICI treatment was associated with a better OS than irinotecan-based chemotherapy [HR 0.54 (95% CI 0.30–0.99); *p* = 0.047)] in patients without peritoneal metastasis. Regarding PD-L1 expression, ICI treatment was associated with significantly shorter OS than irinotecan-based chemotherapy in patients without PD-L1 expression [HR 1.62 (95% CI 1.03–2.55); *p* = 0.037] (Fig. 3).

### Safety outcomes

Hematologic AEs were more common in the irinotecan-based chemotherapy group than in the ICI group (Table 4). Grade 3–4 neutropenia and anemia were reported significantly more frequently in patients with irinotecan-based chemotherapy than in those with ICI treatment [20% versus 2% (*p* < 0.001) and 22% versus 12% (*p* = 0.018), respectively]. Any-grade nausea and diarrhea were reported significantly more frequently in the irinotecan-based chemotherapy group than in the ICI group [19% versus 6% (*p* < 0.001) and 12% versus 5% (*p* = 0.018), respectively]. In the ICI group, four patients (3%) including one grade 3 AE had hypothyroidism and two patients (2%) had adrenal insufficiency. Also, there was one case of grade 3 inflammatory arthritis and one fatal case of fulminant myocarditis in the ICI group.

**Table 4:** Treatment-related adverse events

Toxicity	Immune checkpoint inhibitor (*n* = 127)		Irinotecan-based chemotherapy (*n* = 233)		
	No. of patients (%)		No. of patients (%)		
	Any grade	G3-4	Any grade	G3-4	P-value for any grade/G3-4 AE
**Hematologic**					
Neutropenia	5 (3.9)	2 (1.6)	85 (36.5)	47 (20.2)	< 0.001/<0.001
Febrile neutropenia	-	-	5 (2.1)	5 (2.1)	0.166/0.166
Anemia	77 (60.6)	15 (11.8)	166 (71.2)	51 (21.9)	0.040/0.018
Thrombocytopenia	17 (13.4)	7 (5.5)	51 (21.9)	12 (5.2)	0.049/0.883
**Non-hematologic**					
Nausea	7 (5.5)	1 (0.8)	45 (19.4)	18 (7.8)	< 0.001/0.005
Vomiting	11 (8.7)	5 (3.9)	32 (13.7)	11 (4.7)	0.156/0.730
Mucositis	1 (0.8)	-	7 (3.0)	1 (0.4)	0.173/0.999
Diarrhea	6 (4.7)	1 (0.8)	29 (12.4)	8 (3.4)	0.018/0.168
Fatigue	22 (17.3)	3 (2.4)	45 (19.3)	4 (1.7)	0.643/0.701
AST/ALT elevation	24 (18.9)	3 (2.4)	37 (15.9)	5 (2.1)	0.466/0.999
Hypothyroidism	4 (3.1)	1 (0.8)	-	-	
Adrenal insufficiency	2 (1.6)	-	-	-	

Abbreviations: AST/ALT, Aspartate Aminotransferase/Alanine Aminotransferase; AE, adverse events

### Subsequent treatment

There was no significant difference in the proportion of patients who received subsequent treatment between the two groups (*p* = 0.112). In the ICI group, among 98 patients who developed PD during ICI treatment by the date of data cut-off, 54 (55%) received subsequent fourth-line treatment; FOLFIRI was the most common regimen (29 patients, 54%) followed by irinotecan monotherapy (18 patients, 33%). Among 184 patients who developed PD in the irinotecan-based chemotherapy group, 107 patients (58%) received salvage treatment including 69 patients (64%) with ICI (nivolumab or pembrolizumab). Detailed subsequent chemotherapy regimens are listed in Supplementary Table S3. Obviously, patients who received subsequent treatment showed significantly superior OS compared to those without fourth-line treatment (median OS of 8.9 versus 3.1 months, *p* < 0.001). In total, 161 patients received subsequent treatment and among them, there was no difference in OS between the two groups (median OS 9.2 months in the ICI group versus 8.0 months in the irinotecan-based chemotherapy group; *p* = 0.352).

## Discussion

This multicenter real-world study found no significant difference in terms of PFS and OS between third-line ICI treatment and irinotecan-based chemotherapy after failure of second-line paclitaxel plus ramucirumab therapy in patients with AGC. Previous studies also compared the treatment efficacy of ICI and irinotecan in third or later-line treatment and showed similar results [18–21]. However, our study is the first report of a direct comparison of these two treatment approaches with large number of patients who underwent the same second-line chemotherapy (paclitaxel plus ramucirumab) in real-world practice setting.

Chemotherapy-induced peripheral neuropathy occurs in a significant number of patients after first-line treatment with fluoropyrimidine/platinum doublet and second-line paclitaxel plus ramucirumab. Hence, irinotecan-based chemotherapy is a reasonable treatment option as third-line treatment and is widely used in real-world clinical practice. Anti-PD-1 monoclonal antibodies (nivolumab or pembrolizumab) clearly demonstrated clinical benefits in some patients [9, 10], and nivolumab has been shown to be associated with increased PFS and OS compared with placebo as third or later-line treatment in patients with AGC. However, it is worth noting that OS was increased only by 1.1 months (5.26 versus 4.14 months, respectively, in nivolumab and placebo). ORR in the nivolumab group was 12% and the OS benefit was observed only in patients whose tumor showed response to nivolumab [9, 22]. However, it is difficult for physicians to pick up on specific clinical characteristics that would predict responsiveness to ICI or chemotherapy prior to initiating treatment.

The survival outcomes of our study in the ICI group are comparable to those of ATTRACTION-2 phase III trial comparing nivolumab with placebo [22]. The median PFS, OS, and 1-year OS rate in the ICI group in our study were 2.3 months, 5.5 months and 25% (Fig. 1), respectively, and they were very similar to the results based on a pooled analysis of several phase 3 clinical trials [23, 24]. In both treatment groups of our study, more than 50% of patients received salvage treatment due to tumor progression after third-line treatment; 48% (47/98) were treated with irinotecan-containing chemotherapy in the ICI group, and 38% (69/184) were treated with PD-1 inhibitors in the irinotecan-based chemotherapy group. Long-term survival was observed in some patients; the 2-year OS rate remained high in the ICI group at 20% compared with 10% in the irinotecan-based chemotherapy group. However, since the median follow-up duration was 12.6 months in our study, we need to consider the possibility that this long-term OS rate was somewhat overestimated.

Regarding tumor response to third-line treatment, we expected that cytotoxic treatment would be associated with higher ORR than ICI treatment. However, ORR was significantly higher in the ICI treatment group (11.6%) than in the irinotecan-based chemotherapy group (5.6%) (Table 2). This can be explained by the decrease in ORR when irinotecan was used as later line treatment, in line with other studies [13, 18, 19, 25], and ORR of 13–20% was reported when irinotecan was used as second-line treatment [26–29]. Disease control rate (DCR) was similar between the two groups. Factors negatively associated with PFS were poor PS (ECOG 2/3), weight loss ≥ 10% within 3 months before start of third-line treatment, and non-MSI-H [or proficient MMR (pMMR)] status. In addition to weight loss and non-MSI-H/pMMR status, peritoneal seeding and hyponatremia were significantly associated with poor OS as reported by the ATTRACTION-02 exploratory analysis [30]. Hypoalbuminemia was also significantly associated with poor OS (Table 3).

We performed subgroup analyses in consideration of clinical and molecular factors to identify the characteristics of patients that are associated with benefits of ICI treatment and irinotecan-based cytotoxic chemotherapy. Interestingly, the subgroup analyses revealed that ICI treatment was associated with better OS than irinotecan-based chemotherapy in patients without peritoneal metastasis, while irinotecan-based chemotherapy showed better OS than ICI treatment in patients without PD-L1 expression (Fig. 3).

In this study, the incidences of tumors that are MSI-H/dMMR, EBV-positive, and of PD-L1 ≥ 1% were 5%, 5% and 48%, respectively. MSI-H/dMMR status is a well-known predictive marker for ICI treatment and positive EBV status is also suggested as a predictive biomarker for AGC [31–33]. In our patient cohort, there were 27 patients (7%) whose tumors were MSI-H/dMMR or EBV-positive status. Except for three cases without MSI/MMR status data, MSI-H/dMMR and EBV positivity were mutually exclusive (Supplementary Table S1) as noted in a previous study [34]. In this patient subset, ICI treatment demonstrated significantly superior PFS compared to irinotecan-based chemotherapy (12.7 months versus 2.8 months). This superior PFS was not translated to significant improvement of OS, probably because the number of patients was small and seven out of 16 patients (44%) in the irinotecan-based chemotherapy group further received PD-1 inhibitors as salvage therapy (Fig. 2). Meanwhile, there is no general consensus on the role of PD-L1 in GC (e.g., utility in predicting effectiveness of ICI treatment, type of antibody most appropriate for testing, and cut-off value). Previous ATTRACTION-02 demonstrated better OS of nivolumab than placebo independently of PD-L1 expression [9], whereas a recent study on first-line treatment with nivolumab combined with chemotherapy showed more favorable results in patients with PD-L1 CPS ≥ 5 [2]. Regardless of some limitations of the current study’s PD-L1 data, ICI treatment was associated with worse OS than irinotecan-based chemotherapy in patients with PD-L1 negative tumors (Fig. 3).

As ICI combined with chemotherapy has become a new standard first-line treatment approach for AGC with PD-L1 expression [2–4], although the cut-off values and detection methods of PD-L1 expression vary for individual anti-PD1 agents, a change is expected in the positioning of PD-1 inhibitors as later-line treatment. However, ICI is still thought to be an important later-line treatment option in ICI-naïve tumors with no/low PD-L1 expression or with HER2-positive or claudin 18.2-positive tumors [14–16]. Therefore, we conducted another explorative analysis for patients with tumors with PD-L1-negative and/or HER2-positive tumors. In this subgroup, ICI and irinotecan-based chemotherapy did not show different treatment outcomes (Supplementary Figure S1).

As expected, hematologic AEs of all grades were more common in patients treated with irinotecan-based chemotherapy than in patients treated with ICI. Additionally, severe neutropenia, anemia, and nausea were observed significantly more frequently in the irinotecan-based chemotherapy group than in the ICI group; however, treatment discontinuation due to these AEs was not reported. On the other hand, there was one fatal immune-related AE (myocarditis) in the ICI group.

This study has several limitations. There were some missing values for MSI/MMR status, EBV status, and PD-L1 expression of tumors. Especially, data on accurate PD-L1 expression levels (i.e., the levels of TPS or CPS expression) was lacking and not included in the analysis. Therefore, the exploratory survival analysis for tumors with low PD-L1 expression (i.e., PD-L1 CPS ≤ 5) could not be performed. There is a chance of underestimated AEs considering the retrospective nature of the present study. The dose intensity could not be calculated because data on the dose and administration interval of anticancer drugs were not collected; if these data were collected together, it would be possible to check in detail the effect of dose intensity on treatment outcomes and AEs. Treatment selection was based on the physician’s discretion, suggesting the possibility of selection bias. Finally, a fair comparison of effectiveness outcomes between the two treatment groups was not feasible due to the non-randomized nature of the study.

Overall, no significant difference in survival outcome was observed in the present study which compared ICI and irinotecan-based chemotherapy as the third-line treatment in patients with AGC in the real-world clinical practice. In terms of treatment-related AEs, ICI treatment was much more advantageous than irinotecan-based chemotherapy. ICI might be preferred for patients without peritoneal metastasis, while irinotecan-based chemotherapy may be a more effective option for patients with tumors lacking PD-L1 expression. Considering that the combination of nivolumab and chemotherapy has become a new standard first-line treatment, more studies on the optimal selection of third-line treatment and appropriate sequence are urgently needed.

### Electronic supplementary material

Below is the link to the electronic supplementary material.

Supplementary Material 1

Supplementary Material 2

## Data Availability

The datasets generated during and/or analysed during the current study are available from the corresponding author on reasonable request.

## Abbreviations

ICI: Immune checkpoint inhibitor
AGC: Advanced gastric cancer
OS: Overall survival
GC: Gastric cancer
HER2: Human epidermal growth factor receptor 2
PD-L1: Programmed death-ligand 1
PS: Performance status
anti-PD-1: Anti-programmed death-1
FOLFIRI: 5-fluorouracil, leucovorin and irinotecan
PD: Progressive disease
AEs: Adverse events
RECIST: Response Evaluation Criteria In Solid Tumor
NCI-CTCAE: National Cancer Institute-Common Terminology Criteria for Adverse Events
PFS: Progression-free survival
MSI: Microsatellite instability
MMR: Mismatch repair
EBV: Epstein-Barr virus
IHC: Immunohistochemical
TPS: Tumor proportion score
MSI-H: MSI-high
dMMR: MMR-deficient
CI: Confidence interval
HR: Hazard ratio
ORR: Objective response rate
CR: Complete response
PR: Partial response
pMMR: Proficient MMR
