# Supplementary material for: Real-world outcomes of third-line immune checkpoint inhibitors versus irinotecan-based chemotherapy in patients with advanced gastric cancer: a Korean, multicenter study (KCSG ST22-06)
Source: BMC Cancer. 2024 Feb 23;24:252. doi: 10.1186/s12885-024-11972-w (PMC10885390; doi:10.1186/s12885-024-11972-w)

Supplementary Figure S1. Survival outcomes of immune checkpoint inhibitor (ICI) versus irinotecan-based chemotherapy as third-line treatment for patients with PD-L1-negative and/or HER2-positive tumors (n=157)

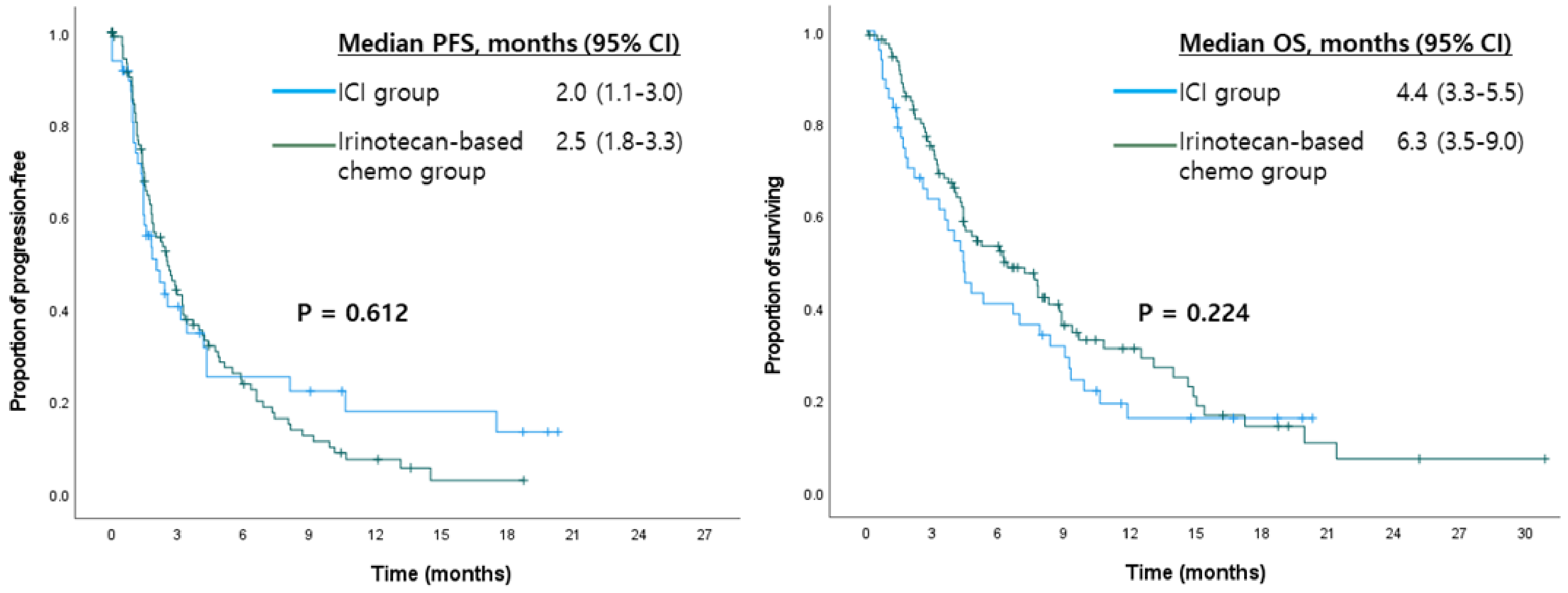

Supplement: Supplementary file 1 — Supplementary Material 1 [file 12885_2024_11972_MOESM1_ESM.pdf]
