# Supplementary material for: Real-world outcomes of third-line immune checkpoint inhibitors versus irinotecan-based chemotherapy in patients with advanced gastric cancer: a Korean, multicenter study (KCSG ST22-06)
Source: BMC Cancer. 2024 Feb 23;24:252. doi: 10.1186/s12885-024-11972-w (PMC10885390; doi:10.1186/s12885-024-11972-w)
Supplement: Supplementary file 2 — Supplementary Material 2 [file 12885_2024_11972_MOESM2_ESM.pdf]

Supplementary Table S1. Clinical factors and outcomes in patients with MMR-D and EBV-positive status (N=27)

Treatment	MSI-H /MMR-D	EBV	PD-L1≥1	Age (years)	Sex	ECOG PS	Peritoneal metastasis	Best response	Subsequent treatment	PFS (M)	OS (M)
Nivolumab	+	-	+	74	F	1	No	CR		15.93+	15.93+
Nivolumab	+	-	-	74	F	2	No	PR		19.83+	19.83+
Nivolumab	+	-	NA	59	M	1	Yes	PR		12.30+	12.30+
Nivolumab	+	-	NA	60	F	1	Yes	SD		7.70+	7.70+
Nivolumab	+	-	NA	62	F	2	Yes	PR	FOLFIRI	4.40	9.67
Nivolumab	+	-	+	67	M	1	No	PR	Irinotecan	12.73	14.50+
Nivolumab	-	+	+	60	M	1	Yes	NE		0.63	0.63
Pembrolizumab	+	-	+	46	F	2	Yes	PD		0.70	1.37
Pembrolizumab	+	-	NA	67	M	1	No	SD	Irinotecan	3.93	6.90
Pembrolizumab	+	-	+	77	M	0	No	CR		15.57	15.57+
Pembrolizumab	-	+	+	76	M	1	No	PR	FOLFIRI	5.73	7.83+
Irinotecan	+	-	+	64	M	1	No	PD	MEDI5752	0.97	27.23+
Irinotecan	+	-	+	73	F	0	No	SD	Pembrolizumab	5.57	13.37+
Irinotecan	-	+	+	44	M	1	Yes	PD		1.20	2.77
Irinotecan	NA	+	NA	80	M	2	No	PD		1.90	12.37
Irinotecan	-	+	+	53	M	1	No	PD		0.77	2.60
FOLFIRI	+	-	NA	63	F	1	Yes	SD		6.47+	6.47+
FOLFIRI	+	-	NA	79	M	1	Yes	NE		0.50	3.23
FOLFIRI	+	-	+	55	M	0	Yes	SD	Pembrolizumab+ Bevituximab	4.20	20.83+
FOLFIRI	-	+	-	52	M	0	Yes	SD	Docetaxel	6.87	13.07
FOLFIRI	-	+	+	69	M	1	No	SD	Nivolumab	2.80	6.73
FOLFIRI	-	+	NA	47	M	1	No	PD	Pembrolizumab	1.83	17.57
FOLFIRI	NA	+	NA	47	M	1	Yes	PD	Docetaxel	0.93	2.90
FOLFIRI	-	+	NA	72	M	1	Yes	SD		1.10	1.10
FOLFIRI	-	+	+	76	M	2	Yes	SD		3.33	6.57
FOLFIRI	-	+	NA	67	M	1	Yes	SD	Pembrolizumab	3.97	7.80
FOLFIRI	NA	+	-	56	M	1	Yes	PD		1.20	1.37

Supplementary Table S2. Univariable analysis of prognostic factors

	PFS			OS		
	HR	95% CI	P value	HR	95% CI	P value
Treatment group						
ICI versus Irinotecan-based chemotherapy (Ref)	0.97	0.76-1.24	0.803	0.97	0.75-1.25	0.787
Age (years)						
≥65 versus <65 (Ref)	0.89	0.69-1.16	0.403	0.77	0.58-1.02	0.068
Age (years)						
≥70) versus <70 (Ref)	0.95	0.68-1.31	0.734	0.78	0.55-1.12	0.180
ECOG PS						
2/3 versus 0/1(Ref)	1.40	0.99-1.98	0.056	2.02	1.46-2.78	<0.001
Sex						
Male versus Female (Ref)	1.03	0.81-1.30	0.815	1.04	0.81-1.34	0.749
Primary site						
GEJ versus Stomach (Ref)	1.86	1.04-3.33	0.037	0.90	0.45-1.83	0.775
Gastrectomy						
Yes versus No (Ref)	0.97	0.77-1.23	0.810	1.11	0.86-1.42	0.423
Weight loss						
Yes versus No (Ref)	1.57	1.22-2.03	<0.001	1.88	1.44-2.46	<0.001
Liver metastasis						
Yes versus No (Ref)	1.11	0.86-1.41	0.428	0.96	0.74-1.26	0.786
Peritoneal metastasis						
Yes versus No (Ref)	1.17	0.90-1.53	1.234	2.29	1.68-3.13	<0.001
HER2 status						
Positive versus Negative (Ref)	1.12	0.79-1.60	0.533	0.83	0.56-1.22	0.345
EBV status						
Positive versus Negative (Ref)	1.58	0.88-2.83	0.126	1.15	0.61-2.17	0.673
MSI/MMR status						
MSI-H/dMMR versus others (Ref)	0.33	0.15-0.70	0.004	0.10	0.03-0.41	0.001
NLR						
≥2.59 versus <2.59 (Ref)	1.14	0.90-1.45	0.26	1.69	1.32-2.17	<0.001
PLR						
≥157.34 versus <157.34 (Ref)	1.19	0.94-1.51	0.14	1.46	1.14-1.87	0.003
Serum sodium (mEq/L)						
<135 versus≥135 (Ref)	1.57	1.16-2.13	0.004	2.19	1.62-2.97	<0.001
Serum albumin (g/dL)						
<3.5 versus ≥3.5 (Ref)	1.22	0.96-1.55	0.102	2.39	1.83-3.11	<0.001
Duration of 2nd line therapy[a]						
<median versus ≥median (Ref)	1.38	1.09-1.74	0.008	1.55	1.21-1.99	0.001

Abbreviations: PFS, progression-free survival; OS, overall survival; HR, hazard ratio; CI, confidence interval; Ref, reference; ECOG, Eastern Cooperative Oncology Group; PS, performance status; MSI/MMR, microsatellite instability/ mismatch repair; NLR, neutrophil to lymphocyte ratio (median, 2.59); PLR, platelet to lymphocyte ratio (median, 157.34)

[a]Duration of 2nd line therapy: the median value was 3.7 months

Supplementary Table S3. Subsequent treatment

ICI group (N=54)		Irinotecan-based chemotherapy group (N=107)	
FOLFIRI	29 (53.7%)	Nivolumab	52 (48.5%)
Irinotecan	18 (33.3%)	Pembrolizumab	15 (14.0%)
FOLFOX	2 (3.7%)	Pembrolizumab/Bavituximab	1 (0.9%)
Trifluridine/Tipiracil	2 (3.7%)	Pembrolizumab/Vactosertib	1 (0.9%)
Pembrolizumab/Bavituximab	1 (1.8%)	Docetaxel/Cisplatin/5-Fluorouracil	1 (0.9%)
S-1	2 (3.7%)	Docetaxel/Cisplatin	2 (1.9%)
		Docetaxel	8 (7.5%)
		Etoposide/Cisplatin	1 (0.9%)
		FOLFOX	1 (0.9%)
		IDX-1197	1 (0.9%)
		Trifluridine/Tipiracil	3 (2.8%)
		Capecitabine	1 (0.9%)
		MEDI5752	1 (0.9%)
		Neratinib	1 (0.9%)
		Regorafenib vs Placebo (clinical trial)	2 (1.9%)
		Rivoceranib	3 (2.8%)
		S-1/Cisplatin	6 (5.6%)
		Trastuzumab/Capecitabine/Cisplatin	1 (0.9%)
		S-1	5 (4.7%)
		Capecitabine/Cisplatin	1 (0.9%)

Abbreviations: FOLFIRI, fluorouracil, leucovorin, irinotecan; FOLFOX, fluorouracil, leucovorin, oxaliplatin
